# LncRNA *C5orf64* polymorphisms (rs12518552 and rs2950218) decreases pulmonary tuberculosis susceptibility

**DOI:** 10.1080/07853890.2025.2523557

**Published:** 2025-06-28

**Authors:** Shilin Xu, Baoping Hu, Dongfeng Zhang, Jing Wang, Xue He, Yongjun He, Yuhe Wang

**Affiliations:** a Department of Clinical Laboratory, The Affiliated Hospital of Xizang Minzu University, Xianyang, China; b Department of Anesthesia, The Affiliated Hospital of Xizang Minzu University, Xianyang, China; c Key Laboratory of Molecular Mechanism and Intervention Research for Plateau Diseases of Tibet Autonomous Region, School of Medicine, Xizang Minzu University, Xianyang, China

**Keywords:** Pulmonary tuberculosis, C5orf64, polymorphisms, genetic susceptibility, stratified analysis

## Abstract

**Background:**

Pulmonary tuberculosis (PTB) remains a significant global health issue, with genetic factors playing a crucial role in susceptibility. Long noncoding RNA (lncRNA) *C5orf64* has been implicated in immune responses and cancer, but its association with PTB risk has not been fully explored.

**Methods:**

Genomic DNA was extracted from peripheral blood samples of 955 participants (474 PTB cases and 481 controls). Rs12518552 and rs2950218 in *C5orf64* were genotyped using the Agena MassARRAY system. Logistic regression analysis was performed to assess the association between these polymorphisms and PTB risk. Stratified analysis was conducted to evaluate the influence of age, gender, and smoking status.

**Results:**

Rs12518552-G (OR = 0.82, *p* = 0.034) and rs2950218-T (OR = 0.77, *p* = 0.012) were associated with a reduced PTB risk. Stratified analysis revealed that rs12518552 was associated with a protective effect against PTB in individuals over 40 years old (OR = 0.73, *p* = 0.024), females (OR = 0.77, *p* = 0.034), and non-smokers (OR = 0.78, *p* = 0.040), and rs2950218 was also associated with a reduced PTB risk in individuals over 40 years old (OR = 0.73, *p* = 0.040), females (OR = 0.72, *p* = 0.046), and non-smokers (OR = 0.72, *p* = 0.011).

**Conclusion:**

*C5orf64* polymorphisms, particularly rs12518552 and rs2950218, are associated with a reduced risk of PTB. These findings suggest that C5orf64 polymorphisms contribute to genetic susceptibility to PTB, with implications for PTB targeted screening and personalized therapeutic strategies.

## Introduction

Pulmonary tuberculosis (PTB), a chronic infectious disease predominantly affecting the lungs, continues to be a major global public health concern [1]. In 2022, tuberculosis (TB) affected approximately 10.6 million people worldwide, including 5.8 million men, 3.5 million women, and 1.3 million children, leading to 1.3 million deaths [2]. China’s TB report data from 2006 to 2020 shows 14.82 million PTB cases, despite a declining incidence rate, indicating a persistent high burden in the country [3]. Factors such as gender, age, urban versus rural residency, and regional differences influence PTB incidence rates variably [3,4]. Several pivotal risk factors are known to exacerbate the incidence of TB, including human immunodeficiency virus (HIV) infection [5], diabetes mellitus [5], undernutrition [5], poverty [6], indoor air pollution [7], and climate change [8]. Despite advances in diagnostic techniques for PTB [9,10], the disease continues to pose challenges in terms of early detection and effective treatment. Genetic factors are increasingly recognized as critical determinants of PTB susceptibility [11,12], making the exploration of genetic contributors essential for personalized prevention and therapeutic strategies.

Long noncoding RNAs (lncRNAs), characterized by their length exceeding 200 nucleotides and lack of protein-coding potential, are gaining recognition for their significant roles in disease pathogenesis [13]. Emerging research highlights the role of lncRNAs in infectious diseases, where they regulate host-pathogen interactions and immune responses. For example, lncRNAs such as *MALAT1* and *NEAT1* have been shown to influence inflammation and autophagy in bacterial and viral infections, underscoring their importance in modulating host defense mechanisms [14,15]. Recent studies have identified unique lncRNA expression profiles in PTB, elucidating their functional roles and the implications of genetic variations within these molecules [16–20]. Among these, C5orf64, a lncRNA, has been implicated in immune regulation through its influence on inflammatory pathways and cytokine signaling. Previous studies suggest that lncRNAs like *C5orf64* modulate the expression of genes critical for host defense mechanisms, potentially affecting macrophage activation and T-cell responses during Mycobacterium TB (*Mtb*) infection [21–24]. *C5orf64* has also been associated with ferroptosis and implicated in lung adenocarcinoma [25], serves as a marker for tumor microenvironment (TME) alterations and mutation patterns [26]. However, the specific role of *C5orf64* in PTB remains underexplored, particularly regarding how its genetic variants might affect susceptibility to infection. Considering the complex immune reactions triggered by *Mtb* [27], it is postulated that polymorphisms in the *C5orf64* gene might disrupt immune regulation and thereby influence an individual’s susceptibility to PTB. Despite advances in understanding PTB pathogenesis, the functional consequences of SNPs in lncRNAs on PTB susceptibility remain poorly defined. Existing studies have largely focused on protein-coding genes, leaving significant gaps in our knowledge of how lncRNA polymorphisms influence host defense [28]. To date, a comprehensive study on the existence of these polymorphisms and their influence on PTB risk has not yet been conducted.

Therefore, this study aims to address these gaps by elucidating the association between *C5orf64* polymorphisms and the risk of developing PTB in a Chinese Han population. By examining genetic variations in *C5orf64*, we seek to enhance the understanding of the genetic basis of PTB, identify novel genetic markers for risk assessment, and facilitate targeted preventive and therapeutic interventions.

## Methods

### Sample collection

**Figure 1.**
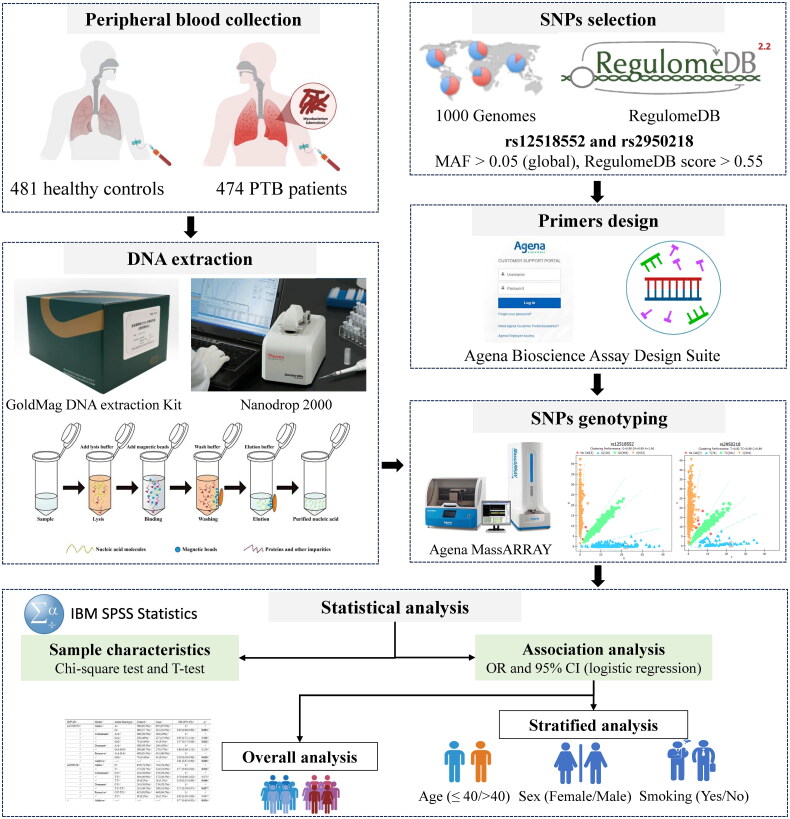
Flowchart of the case-control study on *C5orf64* gene polymorphisms and PTB susceptibility.

This study aims to investigate the association between *C5orf64* polymorphisms and susceptibility to PTB. A visual workflow summarizing the key steps in the study is presented in Figure 1. The required sample size was calculated a priori using G*Power (version 3.1.9.7) to ensure adequate statistical power. Specifically, the calculation was based on an effect size *d* = 0.2, a significance level (α err prob) of 0.05, and a power (1-B err prob) of 0.85, with an allocation ratio (N2/N1) of 1.1. According to these parameters, the required sample size was determined to be 450 for each group. To account for potential attrition or data exclusions, we recruited 474 PTB cases and 481 healthy controls, ensuring robust statistical validity for detecting associations between *C5orf64* gene polymorphisms and PTB risk. A total of 955 participants were randomly recruited from the Affiliated Hospital of Xizang Minzu University. Participants provided 5 mL of peripheral venous blood sample in EDTA tubes and stored at −80 °C until DNA extraction. Cases were defined as individuals with a confirmed diagnosis of active PTB based on clinical symptoms, chest X-ray findings, and positive sputum smear microscopy for *Mtb*. Controls were healthy individuals, free from active (as confirmed by the absence of symptoms and normal chest radiograph findings) or prior TB, and matched to cases by age and gender to reduce confounding effects. Both the cases and controls were of Chinese Han ethnicity. Subjects with a prior history of any TB type, concurrent serious chronic conditions (including diabetes, cardiovascular disease, or malignancies), other chronic respiratory illnesses, immunodeficiency conditions, or those who had recently undergone immunosuppressive treatment were excluded from the study. Additionally, pregnant or breastfeeding women were not included. Demographic details (age, sex, and smoking status) were gathered *via* a questionnaire.

### Ethical statement

The study protocol was reviewed and approved by the Ethics Committee of the Xizang Minzu University (No. 2023-11), and all procedures were conducted in accordance with the Declaration of Helsinki. Written informed consent was obtained from all participants prior to sample collection. Participants were fully informed about the objectives, procedures, and potential risks of this study, and their right to withdraw at any time without consequences.

### Genotyping

Genomic DNA was isolated from whole blood samples utilizing the GoldMag Purification Kit (GoldMag Co. Ltd, Xi’an, China) following the standard protocol. Subsequently, to ensure the quality of the genotyping, a spectrophotometer (NanoDrop 2000, Thermo Scientific, Waltham, MA, USA) was employed to measure the concentration and purity of extracted DNA. Two single nucleotide polymorphisms (SNPs, rs12518552 and rs2950218) in the *C5orf64* gene were selected for the current study. Both SNPs have a minor allele frequency (MAF) greater than 0.05 in the global population, based on data from the 1000 Genomes Project database, ensuring sufficient statistical power for association analysis in diverse cohorts. Meanwhile, these SNPs were identified as potentially functionally significant based on their evaluation in RegulomeDB (https://regulomedb.org/regulome-search/). Other SNPs in the *C5orf64* gene were excluded due to a MAF ≤ 0.05, a RegulomeDB score ≤ 0.55, or a lack of expression quantitative trait locus (eQTL) or a cis-acting eQTL (caQTL) or chromatin accessibility evidence. The polymerase chain reaction (PCR) and single base extension (SBE) primers for these SNPs were designed *via* Agena’s tool and synthesized by Sangon Biotech (Shanghai), with sequences in Supplement Table 1. The PCR thermal conditions were as follows: initial denaturation at 94 °C for 15 min, followed by 45 cycles of (denaturation at 94 °C for 20 s, annealing at 56 °C for 30 s, and extension at 72 °C for 1 min), with a final extension at 72 °C for 3 min. SNP genotyping was carried out on the Agena MassARRAY system per the manufacturer’s instructions, with results visualized *via* Agena Bioscience TYPER software (v4.0).

**Table 1.** Feature analysis of sampled subjects.

Variables		Cases	Control	*p*
Total		474	481	
Age,years	Mean ± SD	39.8 ± 17.8	41.3 ± 16.4	0.184^a^
	>40	210 (44.3%)	228 (47.4%)	0.337^b^
	≤40	264 (55.7%)	253 (52.6%)	
Sex	Male	304 (64.1%)	292 (60.7%)	0.274^b^
	Female	170 (35.9%)	189 (39.3%)	
Smoking	Yes	145 (30.6%)	149 (31.0%)	0.897^b^
	No	329 (69.4%)	332 (69.0%)	

SD: standard deviation. ^a^*p* was calculated using the *t*-test. ^b^*p* was calculated using the chi-square test. *p* < 0.05 is considered statistically significant.

## Statistical analysis

Demographic characteristics of the study cohort were assessed using SPSS (v20). We employed a t-test to compare the mean age differences between the control and case groups, while the Pearson chi-square test was applied to examine the distributional variations in gender, smoking status, and age groups across both groups. Genetic equilibrium within the control group was evaluated using the chi-square test to compare observed and expected genotype frequencies. A *p*-value for Hardy-Weinberg equilibrium (HWE) below 0.05 suggests potential genotyping inaccuracies or population stratification. To evaluate the association between SNPs rs12518552 and rs2950218 and PTB susceptibility, logistic regression analysis was performed to calculate odds ratios (ORs) and 95% confidence intervals (CIs), with adjustments for potential confounders such as age, gender, and smoking status using PLINK software (v1.9) under five genetic models (allele, co-dominant, dominant, recessive, and additive models). Stratified analysis was conducted to assess the influence of age, gender, and smoking status. Forest plots illustrating the significant findings of the stratified analysis were generated using the Sangerbox online platform.

## Results

### Sample characteristics analysis

In the study, a total of 955 participants were enrolled, comprising 474 cases and 481 controls. Table 1 presents a comparative analysis of basic information between the case and control groups. Regarding age distribution, the mean age was 39.8 years with a standard deviation of 17.8 in the case group, and 41.3 years with a standard deviation of 16.4 in the control group. The comparison of age between groups revealed no significant difference (*p* = 0.184). When stratified by age, 44.3% of the cases and 47.4% of the controls were above 40 years old, while 55.7% of the cases and 52.6% of the controls were 40 years old or younger. This stratified analysis also showed no statistically significant difference in age distribution (*p* = 0.337). In terms of gender composition, the case group included 64.1% males and 35.9% females, whereas the control group comprised 60.7% males and 39.3% females. The gender distribution comparison did not yield a statistically significant difference (*p* = 0.274). Regarding smoking habits, 30.6% of the cases and 31.0% of the controls reported being smokers. The prevalence of smoking in both groups was similar, and the difference was not statistically significant (*p* = 0.897).

### Two genetic variants in the *C5orf64* gene

The features of the two *C5orf64* gene variants examined in this research are delineated in Table 2. The SNP rs12518552 is located at chromosome 5 position 61675292, with a mutation of allele A (wild-type allele) to allele G (variant allele). It is situated within an intronic region and does not appear to deviate from HWE (*p* = 0.697). The MAF in cases is 0.330, slightly lower than in controls, where it is 0.377. This SNP is predicted to have a multifaceted role, including being an eQTL or a caQTL, as well as being associated with transcription factor (TF) binding sites and chromatin accessibility peaks, suggesting a potential regulatory function in gene expression. The second SNP, rs2950218, is positioned 500 base pairs downstream of the gene at chromosome 5 position 61731215, with a mutation of allele C (wild-type allele) to allele T (variant allele). It also adheres to HWE with a *p*-value of 0.999. The MAF for this SNP is 0.236 in cases and 0.287 in controls, indicating a relatively low frequency of the minor allele in both groups. It is predicted to have regulatory potential, as it is implicated in eQTL/caQTL mechanisms, TF binding, and chromatin state modulation, which could influence gene expression patterns.

**Table 2.** Overview of the two selected variants in the *C5orf64* gene.

SNP-ID	Position	Allele A/B	Role	HWE-*p*	MAF-Case	MAF-Control	Function prediction
rs12518552	5: 61675292	G/A	intron	0.697	0.330	0.377	eQTL/caQTL + TF binding / chromatin accessibility peak
rs2950218	5: 61731215	T/C	500B downstream	0.999	0.236	0.287	eQTL/caQTL + TF binding / chromatin accessibility peak

SNP: single nucleotide polymorphism; A/B: minor (variant)/major (wild-type) allele; MAF: minor allele frequency; HWE: Hardy-Weinberg equilibrium; eQTL: expression quantitative trait locus; caQTL: cis-acting eQTL; TF: transcription factor. *p* < 0.05 indicates statistical significance.

### Overall analysis of the association of polymorphisms with PTB risk

We analyzed the association between polymorphisms of *C5orf64* and PTB risk under different genetics models using logistic regression, as shown in Table 3. For the rs12518552 polymorphism, the G allele was less frequent in cases compared to controls (33.0% vs. 37.7%), and indicated a reduced risk of PTB compared with the A allele (OR = 0.82, 95% CI: 0.68–0.99, *p* = 0.034). In the codominant model, the G/G genotype was associated with a lower risk of PTB (G/G vs. A/A: OR = 0.57, 95% CI: 0.37–0.88, *p* = 0.012). The recessive model also showed a significant reduction in risk for the G/G genotype (G/G vs. A/A-G/A: OR = 0.59, 95% CI: 0.39–0.88, *p* = 0.010). The log-additive model yielded a similar result, with an OR of 0.81 (95% CI: 0.67–0.99, *p* = 0.035).

**Table 3.** Association of *C5orf64* gene polymorphisms with PTB risk under various genetic models.

SNP-ID	Model	Allele/Genotype	Control	Case	OR (95% CI)	*p*
rs12518552	Allelic	A	596 (62.3%)	635 (67.0%)	1	
		G	360 (37.7%)	313 (33.0%)	0.82 (0.68-0.99)	**0.034**
	Codominant	A/A	188 (39.3%)	204 (43%)	1	
		G/A	220 (46%)	227 (47.9%)	0.95 (0.72-1.24)	0.698
		G/G	70 (14.6%)	43 (9.1%)	0.57 (0.37-0.88)	**0.012**
	Dominant	A/A	188 (39.3%)	204 (43%)	1	
		G/A-G/G	290 (60.7%)	270 (57%)	0.86 (0.66-1.11)	0.250
	Recessive	A/A-G/A	408 (85.4%)	431 (90.9%)	1	
		G/G	70 (14.6%)	43 (9.1%)	0.59 (0.39-0.88)	**0.010**
	Additive	–	–	–	0.81 (0.67-0.99)	**0.035**
rs2950218	Allelic	C	676 (71.3%)	724 (76.4%)	1	
		T	272 (28.7%)	224 (23.6%)	0.77 (0.63-0.94)	**0.012**
	Codominant	C/C	241 (50.8%)	276 (58.2%)	1	
		T/C	194 (40.9%)	172 (36.3%)	0.78 (0.60-1.02)	0.073
		T/T	39 (8.2%)	26 (5.5%)	0.59 (0.35-0.99)	**0.046**
	Dominant	C/C	241 (50.8%)	276 (58.2%)	1	
		T/C-T/T	233 (49.2%)	198 (41.8%)	0.75 (0.58-0.97)	**0.027**
	Recessive	C/C-T/C	435 (91.8%)	448 (94.5%)	1	
		T/T	39 (8.2%)	26 (5.5%)	0.65 (0.39-1.09)	0.097
	Additive	–	–	–	0.77 (0.63-0.95)	**0.014**

PTB: pulmonary tuberculosis; SNP: single nucleotide polymorphism; OR: odds ratio; CI: confidence interval. OR and 95% CI were determined through logistic regression, taking into account potential confounders. Statistical significance is indicated by bold *p* < 0.05.

For rs2950218, the frequency of the T allele in cases was also less compared to controls (23.6% vs. 28.7%), which was associated with a reduced risk of PTB (OR = 0.77, 95% CI: 0.63–0.94, *p* = 0.012). In the codominant model, the T/T genotype was found to have a reduced risk of PTB (OR = 0.59, 95% CI: 0.35–0.99, *p* = 0.046). The dominant model indicated a significant association between the T/C-T/T genotypes and the risk of PTB (OR = 0.75, 95% CI: 0.58–0.97, *p* = 0.027). The log-additive model supported these findings with an OR of 0.77 (95% CI: 0.63–0.95, *p* = 0.014). However, the recessive model did not show a significant effect.

### Stratified analysis of the association of polymorphisms with PTB risk

**Figure 2.**
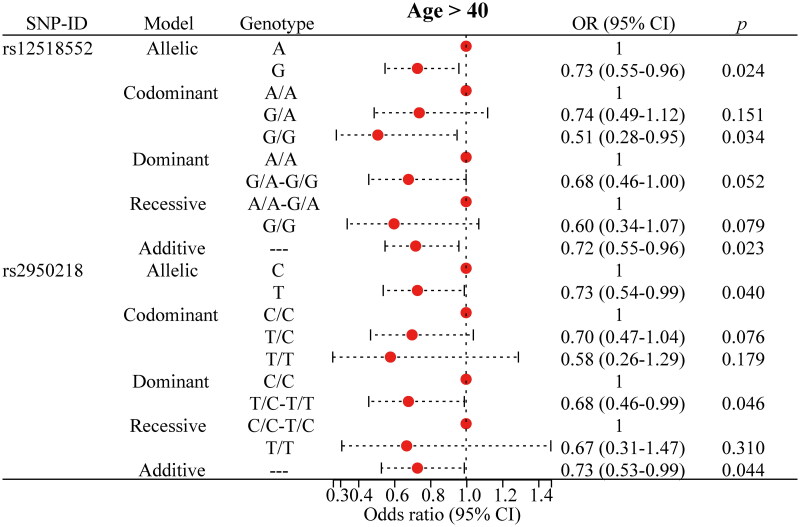
Significant findings between *C5orf64* polymorphisms and PTB risk in age > 40 years population. PTB: pulmonary tuberculosis; SNP: single nucleotide polymorphism; OR: odds ratio; CI: confidence interval. The circle represents the OR value. The horizontal lines represented the study-specific 95% CI. OR (95% CI) calculated via logistic regression, adjusted for confounders. *p* < 0.05 indicates statistical significance.

**Figure 3.**
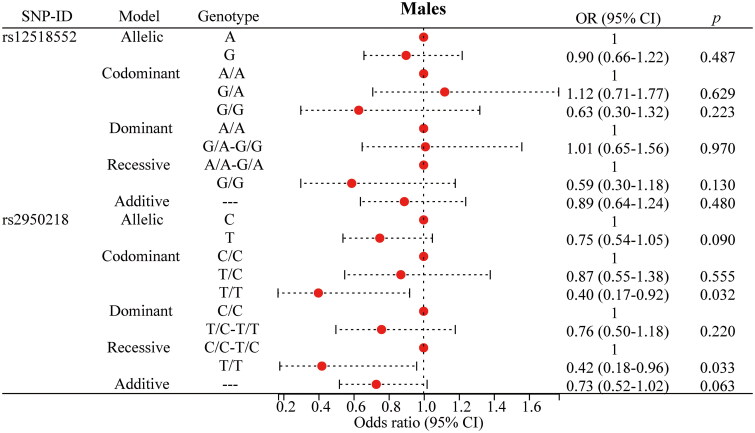
Significant findings between *C5orf64* polymorphisms and PTB risk in males. PTB: pulmonary tuberculosis; SNP: single nucleotide polymorphism; OR: odds ratio; CI: confidence interval. The circle represents the OR value. The horizontal lines represented the study-specific 95% CI. OR (95% CI) calculated via logistic regression, adjusted for confounders. *p* < 0.05 indicates statistical significance.

**Figure 4.**
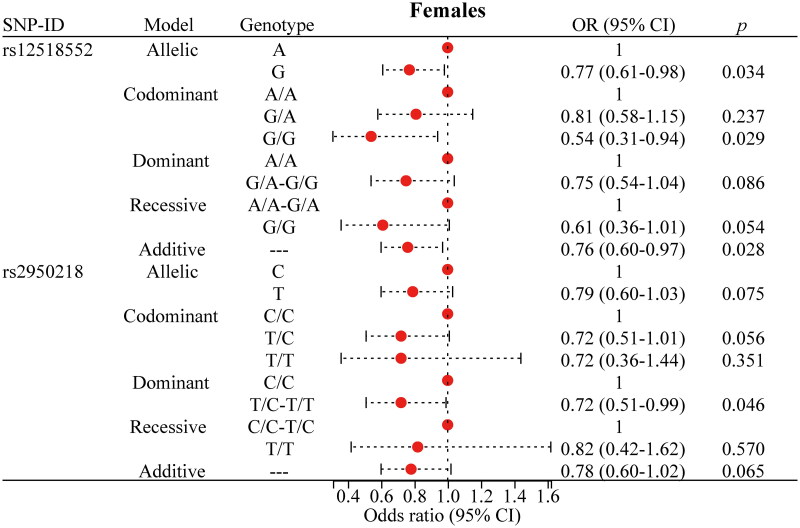
Significant findings between *C5orf64* polymorphisms and PTB risk in females. PTB: pulmonary tuberculosis; SNP: single nucleotide polymorphism; OR: odds ratio; CI: confidence interval. The circle represents the OR value. The horizontal lines represented the study-specific 95% CI. OR (95% CI) calculated via logistic regression, adjusted for confounders. *p* < 0.05 indicates statistical significance.

**Figure 5.**
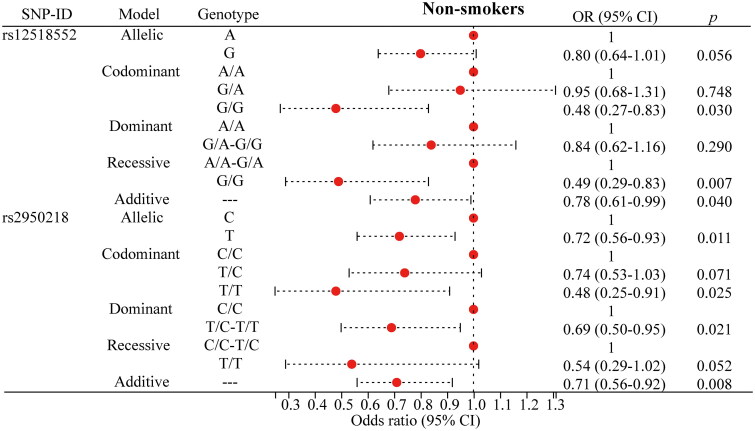
Significant findings between *C5orf64* polymorphisms and PTB risk in non-smokers. PTB: pulmonary tuberculosis; SNP: single nucleotide polymorphism; OR: odds ratio; CI: confidence interval. The circle represents the OR value. The horizontal lines represented the study-specific 95% CI. OR (95% CI) calculated via logistic regression, adjusted for confounders. *p* < 0.05 indicates statistical significance.

To mitigate the influence of age, gender, and smoking on the association between the SNP and the risk of PTB, we performed a stratified analysis, as presented in Figures 2–5 and detailed in Supplementary Table 2. For rs12518552, significant associations with a decreased risk of PTB were identified among individuals aged over 40 years (Figure 2), males (Figure 3) and non-smokers (Figure 5). Specifically, the G allele compared to the A allele showed an OR of 0.73 (*p* = 0.024) in the over-40 years group, 0.77 (*p* = 0.034) in males, and 0.78 (*p* = 0.040) in non-smokers. The G/G genotype versus the AA genotype yielded ORs of 0.51 (*p* = 0.034), 0.54 (*p* = 0.029), and 0.48 (*p* = 0.030) respectively, across these strata. The additive model consistently supported these findings with ORs of 0.72 (*p* = 0.023), 0.76 (*p* = 0.028), and 0.78 (*p* = 0.040). Regarding rs2950218, the T allele compared to the C allele was associated with a reduced risk of PTB, with ORs of 0.73 (*p* = 0.040, Figure 2) in the over-40 years group and 0.72 (*p* = 0.011, Figure 5) in non-smokers. The T/T genotype showed significant associations with a decreased risk of PTB in females (OR = 0.40, *p* = 0.032, Figure 4) and non-smokers (OR = 0.48, *p* = 0.025, Figure 5). The dominant model, comparing T/C-T/T to C/C, yielded ORs of 0.68 (*p* = 0.046, Figure 2) in individuals over 40 years, 0.72 (*p* = 0.046, Figure 3) in males, 0.42 (*p* = 0.033, Figure 4) in females, and 0.69 (*p* = 0.021, Figure 5) in non-smokers. the additive model supported these results with ORs of 0.73 (*p* = 0.044, Figure 2) in those over 40 years and 0.71 (*p* = 0.008, Figure 5) in non-smokers. Moreover, these stratified analyses revealed significant demographic-specific effects of *C5orf64* polymorphisms. For instance, the G allele of rs12518552 showed a stronger protective effect against PTB in females (OR = 0.77, 95% CI: 0.61–0.98, *p* = 0.34) compared to males, while the protective role of the T allele of rs2950218 was more pronounced in non-smokers (OR = 0.72, 95% CI: 0.56–0.93, *p* = 0.011). These findings underscore the importance of demographic factors in genetic susceptibility to PTB, offering novel insights into personalized risk stratification.

## Discussion

This study investigated the association between two polymorphisms in the *C5orf64* gene and the risk of PTB. The statistical analyses demonstrated significant differences in the frequency of the G allele of rs12518552 and the T allele of rs2950218 between cases and controls. These differences underscore the protective effects of these alleles and their potential utility as genetic markers for PTB susceptibility. Moreover, the results indicated that the G allele of rs12518552 and the T allele of rs2950218 in *C5orf64* were associated with a reduced risk of PTB. These findings were consistent across various genetic models and were particularly pronounced in specific subgroups, such as individuals over 40 years old, males, and non-smokers. The stratified analysis further supported these associations, suggesting that these *C5orf64* polymorphisms may play a role in modulating PTB susceptibility.

The *C5orf64* gene, also known as LINC03122, is a non-coding RNA (ncRNA) located on chromosome 5 at position 5q12.1. ncRNAs are known to participate in a myriad of biological processes, including responses to *Mtb* infection [29]. Many studies have highlighted the regulatory role of lncRNAs in gene expression during the host immune response to *Mtb* [30,31]. Recent studies have indicated that the lncRNA *C5orf64* is correlated with the abundance of immune cells [26]. Additionally, the progression of lung adenocarcinoma is thought to be influenced by the lncRNA C5orf64/miR-582-5p/NDRG2/TLR2 axis [32]. Moreover, bioinformatics analysis has revealed a correlation between *C5orf64* and overall survival in colorectal cancer patients [33]. However, the association between *C5orf64* genetic polymorphisms and PTB risk has not been extensively explored. Our study identified associations between *C5orf64* polymorphisms (rs12518552 and rs2950218) and a reduced risk of PTB. The findings of this study have significant translational potential. The identified SNPs could serve as biomarkers for PTB susceptibility, aiding in the development of targeted genetic screening programs. Furthermore, these insights could inform therapeutic strategies, such as designing interventions that modulate the activity of *C5orf64* to enhance host immunity. Integrating these genetic markers into public health initiatives could improve early detection and prevention efforts, especially in high-risk populations.

Given that both SNPs are predicted to influence gene expression through mechanisms such as eQTL/caQTL, TF binding, and chromatin state modulation, they may affect the immune response to *Mtb*. The protective effects of the identified polymorphisms in *C5orf64* may be attributed to their regulatory roles in immune pathways. The G allele of rs12518552 is hypothesized to enhance the transcriptional activity of *C5orf64*, leading to upregulated expression of genes involved in macrophage activation and cytokine production. Similarly, the T allele of rs2950218 may modulate splicing or stability of the lncRNA, influencing the downstream immune response to *Mtb*. Further functional studies are warranted to elucidate these mechanisms and validate their role in PTB pathogenesis.

Furthermore, our stratified analysis demonstrated that rs12518552 showed a stronger protective effect in individuals over 40 years old, males, and non-smokers. Similarly, rs2950218 exhibited a reduced risk in individuals over 40 years old, females, and non-smokers. These findings suggest that the genetic predisposition conferred by these SNPs may interact with age, gender, and smoking status to influence PTB susceptibility. Aging is known to bring immune system changes, including changes in the composition and function of immune cells [34,35]. Additionally, gender-specific immune responses, which have been documented to exhibit distinct patterns of immune activation and cytokine production [35,36], may modulate the impact of these SNPs on PTB risk. The association between smoking status and PTB risk is well-established, with smokers having an increased risk of developing the disease [37]. These genetic variants are more noticeable in non-smokers, where the negative impact of smoking on immunity is not present. Smoking is known to cause damage to the respiratory tract and to weaken immune responses, which could potentially mask the protective effects of these alleles [38]. These findings underscore the complexity of genetic susceptibility to PTB and the importance of considering demographic and environmental factors in genetic studies. Future research should aim to elucidate the underlying biological mechanisms and their interactions with *C5orf64* polymorphisms to enhance our understanding of PTB susceptibility.

Despite the significant findings, the study has several limitations. First, the sample size, although substantial, may not be fully representative of the broader population, potentially limiting the generalizability of the findings. Second, the study was observational and cannot establish causality. Further functional studies are needed to understand the mechanisms by which these SNPs influence PTB susceptibility. Third, the study focused on two SNPs in the *C5orf64* gene, and other genetic variations within or outside the gene could also contribute to PTB risk. Lastly, environmental factors and interactions with other genetic loci were not fully explored in this study, which could be important in the context of PTB susceptibility.

## Conclusion

In conclusion, our study identifies two polymorphisms (rs12518552 and rs2950218) in the *C5orf64* gene that are associated with a reduced risk of PTB. These findings highlight the potential role of genetic factors in modulating PTB susceptibility and underscore the importance of considering genetic variability in the development of personalized approaches for PTB prevention and treatment. Further research should focus on functional studies to elucidate the biological mechanisms underlying the protective effects of rs12518552 and rs2950218 in *C5orf64*. These studies could include transcriptomic and epigenetic analyses to determine how these polymorphisms influence gene expression and immune regulation during *Mtb* infection. Additionally, exploring their interactions with other genetic or environmental factors may provide a more comprehensive understanding of PTB susceptibility.

## Supplementary Material

Supplement Table.xlsx

## Data Availability

All data included in this study are available upon request by contact with the corresponding author.

## References

[1] Furin J, Cox H, Pai M. Tuberculosis. Lancet. 2019;393(10181):1642–1656. doi:10.1016/S0140-6736(19)30308-3. 30904262

[2] Bagcchi S. WHO’s Global Tuberculosis Report 2022. Lancet Microbe. 2023;4(1):e20. doi:10.1016/S2666-5247(22)00359-7. 36521512

[3] Dong Z, Wang Q-Q, Yu S-C, et al. Age-period-cohort analysis of pulmonary tuberculosis reported incidence, China, 2006-2020. Infect Dis Poverty. 2022;11(1):85. doi:10.1186/s40249-022-01009-4. 35902982 PMC9331155

[4] Dong Z, Yao HY, Yu SC, et al. Changes in notified incidence of pulmonary tuberculosis in China. Biomed Environ Sci. 2023;36(2):117–126.doi: 10.3967/bes2023.015. 36861190

[5] Silva DR, Muñoz-Torrico M, Duarte R, et al. Risk factors for tuberculosis: diabetes, smoking, alcohol use, and the use of other drugs. J Bras Pneumol. 2018;44(2):145–152. doi:10.1590/s1806-37562017000000443. 29791552 PMC6044656

[6] Pathak D, Vasishtha G, Mohanty SK. Association of multidimensional poverty and tuberculosis in India. BMC Public Health. 2021;21(1):2065. doi:10.1186/s12889-021-12149-x. 34763696 PMC8582202

[7] Patel V, Foster A, Salem A, et al. Long-term exposure to indoor air pollution and risk of tuberculosis. Indoor Air. 2021;31(3):628–638. doi:10.1111/ina.12756. 33016379 PMC9580027

[8] Kharwadkar S, Attanayake V, Duncan J, et al. The impact of climate change on the risk factors for tuberculosis: a systematic review. Environ Res. 2022;212(Pt C):113436. doi:10.1016/j.envres.2022.113436. 35550808

[9] Suárez I, Fünger SM, Kröger S, et al. The diagnosis and treatment of tuberculosis. Dtsch Arztebl Int. 2019;116(43):729–735. doi:10.3238/arztebl.2019.0729. 31755407

[10] Tiberi S, Du Plessis N, Walzl G, et al. Tuberculosis: progress and advances in development of new drugs, treatment regimens, and host-directed therapies. Lancet Infect Dis. 2018;18(7):e183–e98. doi:10.1016/S1473-3099(18)30110-5. 29580819

[11] Seshadri C, Sutherland JS, Lindestam Arlehamn CS, et al. Editorial: exploring immune variability in susceptibility to tuberculosis infection in humans. Front Immunol. 2021;12:830920. doi:10.3389/fimmu.2021.830920. 35069606 PMC8777100

[12] Chen R, Wang X, Li Z, et al. Human Toll-like receptor 2 genetic polymorphisms with tuberculosis susceptibility: a systematic review and meta-analysis. Cytokine. 2023;172:156405. doi:10.1016/j.cyto.2023.156405. 37883839

[13] Bridges MC, Daulagala AC, Kourtidis A. LNCcation: lncRNA localization and function. J Cell Biol. 2021;220(2):e202009045. doi:10.1083/jcb.202009045. PMC7816648 33464299

[14] Cheng Y, Liang Y, Tan X, et al. Host long noncoding RNAs in bacterial infections. Front Immunol. 2024;15:1419782. doi:10.3389/fimmu.2024.1419782. 39295861 PMC11408731

[15] Shin JJ, Park J, Shin HS, et al. Roles of lncRNAs in NF-κB-mediated macrophage inflammation and their implications in the pathogenesis of human diseases. Int J Mol Sci. 2024;25(5):2670. doi:10.3390/ijms25052670. 38473915 PMC10931578

[16] Ye T, Zhang J, Zeng X, et al. LncRNA CCAT1 is overexpressed in tuberculosis patients and predicts their survival. Immun Inflamm Dis. 2022;10(2):218–224. doi:10.1002/iid3.565. 34847295 PMC8767507

[17] Qu Y, Jiang D, Liu M, et al. LncRNA DANCR restrained the survival of mycobacterium tuberculosis H37Ra by sponging miR-1301-3p/miR-5194. Front Microbiol. 2023;14:1119629. doi:10.3389/fmicb.2023.1119629. 37125193 PMC10133511

[18] Liu L, Yu Z, Ma Q, et al. LncRNA NR_003508 suppresses mycobacterium tuberculosis-induced programmed necrosis via sponging miR-346-3p to regulate RIPK1. Int J Mol Sci. 2023;24(9):8016. doi:10.3390/ijms24098016. PMC10179217 37175724

[19] Liu G, Xia R, Wang Q, et al. Significance of LncRNA CASC8 genetic polymorphisms on the tuberculosis susceptibility in Chinese population. J Clin Lab Anal. 2020;34(6):e23234. doi:10.1002/jcla.23234. 32034808 PMC7307370

[20] Yan H, Liu G, Liang Y, et al. Up-regulated long noncoding RNA AC007128.1 and its genetic polymorphisms associated with Tuberculosis susceptibility. Ann Transl Med. 2021;9(12):1018–1018. doi:10.21037/atm-21-2724. 34277818 PMC8267308

[21] Chen Z-L, Wei L-L, Shi L-Y, et al. Screening and identification of lncRNAs as potential biomarkers for pulmonary tuberculosis. Sci Rep. 2017;7(1):16751. doi:10.1038/s41598-017-17146-y. 29196714 PMC5711916

[22] Dong J, Song R, Shang X, et al. Identification of important modules and biomarkers in tuberculosis based on WGCNA. Front Microbiol. 2024;15:1354190. doi:10.3389/fmicb.2024.1354190. 38389525 PMC10882270

[23] Huang G, Wu X, Ji X, et al. LncRNA SNHG16 inhibits intracellular *M. tuberculosis* growth involving cathelicidin pathway, autophagy, and effector cytokines production. ACS Omega. 2024;9(42):43115–43128. doi:10.1021/acsomega.4c07053. 39464459 PMC11500371

[24] Kotey SK, Tan X, Kinser AL, et al. Host long noncoding RNAs as key players in mycobacteria-host interactions. Microorganisms. 2024;12(12):2656. doi:10.3390/microorganisms12122656. 39770858 PMC11728548

[25] Lu L, Liu LP, Zhao QQ, et al. Identification of a ferroptosis-related LncRNA Signature as a novel prognosis model for lung adenocarcinoma. Front Oncol. 2021;11:675545. doi:10.3389/fonc.2021.675545. 34249715 PMC8260838

[26] Pang Z, Chen X, Wang Y, et al. Long non-coding RNA C5orf64 is a potential indicator for tumor microenvironment and mutation pattern remodeling in lung adenocarcinoma. Genomics. 2021;113(1 Pt 1):291–304. doi:10.1016/j.ygeno.2020.12.010. 33309768

[27] Carabalí-Isajar ML, Rodríguez-Bejarano OH, Amado T, et al. Clinical manifestations and immune response to tuberculosis. World J Microbiol Biotechnol. 2023;39(8):206. doi:10.1007/s11274-023-03636-x. 37221438 PMC10205569

[28] Arunima A, van Schaik EJ, Samuel JE. The emerging roles of long non-coding RNA in host immune response and intracellular bacterial infections. Front Cell Infect Microbiol. 2023;13:1160198. doi:10.3389/fcimb.2023.1160198. 37153158 PMC10160451

[29] Wei L, Liu K, Jia Q, et al. The roles of host noncoding RNAs in *Mycobacterium tuberculosis* infection. Front Immunol. 2021;12:664787. doi:10.3389/fimmu.2021.664787. 34093557 PMC8170620

[30] Zhang X, Chen C, Xu Y. Long non-coding RNAs in tuberculosis: from immunity to biomarkers. Front Microbiol. 2022;13:883513. doi:10.3389/fmicb.2022.883513. 35633669 PMC9130765

[31] Fathizadeh H, Hayat SMG, Dao S, et al. Long non-coding RNA molecules in tuberculosis. Int J Biol Macromol. 2020;156:340–346. doi:10.1016/j.ijbiomac.2020.04.030. 32283111

[32] Sun L, Li W, Zhao Z, et al. Identification of a necroptosis-related prognostic signature and associated regulatory axis in lung adenocarcinoma. Int J Genomics. 2023;2023:8766311–8766320. doi:10.1155/2023/8766311. 37965055 PMC10643042

[33] Herrera-Orozco H, García-Castillo V, López-Urrutia E, et al. Somatic copy number alterations in colorectal cancer lead to a differentially expressed ceRNA Network (ceRNet). Curr Issues Mol Biol. 2023;45(12):9549–9565. doi:10.3390/cimb45120597. 38132443 PMC10742218

[34] Ciarambino T, Para O, Giordano M. Immune system and COVID-19 by sex differences and age. Womens Health. 2021;17:17455065211022262. doi: 10.1177/17455065211022262. PMC8188967 34096383

[35] Lewis ED, Wu D, Meydani SN. Age-associated alterations in immune function and inflammation. Prog Neuropsychopharmacol Biol Psychiatry. 2022;118:110576. doi:10.1016/j.pnpbp.2022.110576. 35588939

[36] Dunn SE, Perry WA, Klein SL. Mechanisms and consequences of sex differences in immune responses. Nat Rev Nephrol. 2024;20(1):37–55. doi:10.1038/s41581-023-00787-w. 37993681

[37] Feng Y, Xu Y, Yang Y, et al. Effects of smoking on the severity and transmission of pulmonary tuberculosis: a hospital-based case control study. Front Public Health. 2023;11:1017967. doi:10.3389/fpubh.2023.1017967. 36778540 PMC9909179

[38] Saint-André V, Charbit B, Biton A, et al. Smoking changes adaptive immunity with persistent effects. Nature. 2024;626(8000):827–835. doi:10.1038/s41586-023-06968-8. 38355791 PMC10881394

